# Metabolic and Regulatory Pathways Involved in the Anticancer Activity of Perillyl Alcohol: A Scoping Review of In Vitro Studies

**DOI:** 10.3390/cancers16234003

**Published:** 2024-11-29

**Authors:** Ana Carolina Batista Brochado, Júlia Alves de Moraes, Bruna Rodrigues de Oliveira, Victor Hugo De Souza Lima, Eric Domingos Mariano, Sachin Karande, Tea Romasco, Paulo Emilio Correa Leite, Carlos Fernando Mourão, Gutemberg Gomes Alves

**Affiliations:** 1 Post-Graduation Program in Science & Biotechnology, Institute of Biology, Fluminense Federal University, Niteroi 24220-900, Brazil; 2 Clinical Research Unit, Antonio Pedro University Hospital, Fluminense Federal University, Niteroi 24020-140, Brazil; 3 R-Crio Criogenia S/A, Campinas 13098-324, Brazil; 4 Dental Research Division, Department of Periodontology and Oral Implantology, Fluminense Federal University, Niteroi 21941-617, Brazil; 5 Division of Dental Research Administration, Tufts University School of Dental Medicine, Boston, MA 02111, USA; 6 Department of Medical, Oral and Biotechnological Sciences, Center for Advanced Studies and Technology (CAST), “G. d’Annunzio” University of Chieti-Pescara, 66100 Chieti, Italy; 7 Department of Basic and Clinical Translational Sciences, Tufts University School of Dental Medicine, Boston, MA 02111, USA

**Keywords:** perillyl alcohol, cancer, regulatory pathway, metabolic pathway, in vitro studies

## Abstract

Background/Objectives: Perillyl alcohol (POH), a plant-derived compound, has demonstrated anti-tumor activity across various human cancers. Understanding the regulatory pathways through which POH exerts its effects is crucial for identifying new therapeutic opportunities and exploring potential drug repositioning strategies. Therefore, this scoping review aims to provide a comprehensive overview of the metabolic and regulatory pathways involved in the anticancer effects of POH, based on in vitro evidence. Methods: Following the PRISMA-ScR 2018 guidelines, a systematic search was conducted in the PUBMED, Web of Science, and Scopus databases. Results: A total of 39 studies were included, revealing that POH exerts its biological effects by modulating several pathways, including the regulation of cyclins, CDKs, and p21, thereby affecting cell cycle progression. It inhibits growth and promotes cell death by attenuating AKT phosphorylation, reducing PARP-1 activity, increasing caspase activity and the FAS receptor and its ligand FASL. Additionally, POH reduces ERK phosphorylation, inhibits RAS protein isoprenylation, and decreases Na/K-ATPase activity. Conclusions: In conclusion, this review delineates the key regulatory pathways responsible for mediating the biological effects of POH in cancer.

## Simple Summary

Perillyl alcohol is a natural compound derived from plants, and it has shown promise as an anti-cancer agent in various types of cancer. This review explores how POH affects cancer cells by examining its impact on metabolic and regulatory pathways, such as those involved in cell cycle regulation and apoptosis. By understanding these mechanisms, researchers can identify new opportunities for cancer treatment and consider POH as a candidate for drug repurposing. The findings from this research may help develop novel therapies that are more effective and potentially less toxic, providing new avenues for cancer treatment.

## 1. Introduction

Cancer continues to be one of the most significant health threats worldwide. In 2022, there were an estimated 20 million new diagnoses of cancer and 9.7 million deaths. Approximately 1 in 5 people will develop cancer in their lifetime, with the number of cases expected to grow 77% by 2050, reaching more than 35 million new cases of cancer [1]. The increasing global cancer burden is a result of both population aging and growth. The Anthropocene era brings new challenges due to increased human impact on environmental factors, which, along with changes in diet and lifestyle, increase the exposure to risk factors, many of which are associated with socioeconomic development [2].

In the late 1940s, conventional chemotherapy was introduced, which includes alkylating agents, antimetabolites, natural products, hormones, and steroids. This method lacks specificity as it eliminates fast-growing cancer cells but also affects fast-growing normal cells in the body. Despite this, cytotoxic chemotherapy is widely used to this day [3]. Targeted therapies began to surface with the premise that cancer cells rely on specific targets for their survival during the late 1980s. A notable example of successful targeted therapy is imatinib mesylate (Gleevec), an inhibitor of the BCR-ABL tyrosine kinase, which catalyzed the advancement of subsequent targeted treatments [2]. Another successful targeted therapy is immunotherapy with monoclonal antibodies. Immune checkpoint inhibitors do not directly kill cancer cells; they mobilize the immune system to kill these cells. This therapy has been successful in some cases, even in metastatic cancer. However, there are significant concerns regarding long-term toxicity [4]. Additionally, chimeric antigen receptor (CAR-T) targeted cell therapy has produced remarkable clinical responses in some hematological malignancies but is less effective in solid tumors and is limited by serious toxicities, antigen escape, and restricted trafficking [5].

The rapid expansion of the cancer therapeutic arsenal has resulted in enormous complexity in treatment and regulatory frameworks. The high cost of many new medicines has created significant challenges in the accessibility of cancer treatment for patients and healthcare systems [6]. The rapidly changing treatment landscape, combined with marginal clinical gains and high costs for many therapies, creates an immensely challenging environment for national governments, policymakers, regulators, private payers, patients, and clinicians around the world [7]. Therefore, drug repurposing is a good strategy to try to decrease the cost of antitumor drugs.

Drug repurposing involves three stages: identifying disease targets (hypothesis generation), testing drug efficacy using in vitro and in vivo models, and advancing to clinical trials. The initial stage is pivotal as hypothesis generation forms the foundation of any drug repurposing effort. In oncology, historically, drug repurposing has mainly stemmed from understanding disease pathways or serendipitous discoveries. Therefore, developing innovative strategies to align existing drugs with new applications could enhance the effectiveness of drug repurposing initiatives [8].

Natural products have been crucial for the advancement of cancer treatments. Research on anti-cancer medication employs natural products because of their structural diversity, novel mechanisms of action, potential as drug leads, role in combination therapies, inspiration for synthetic chemistry, and biological activity, which has less toxicity [9]. (R)-(+)-Limonene is a common natural monocyclic monoterpene, constituting over 90% of the orange peel oil, as well as being found in oils of lavender, peppermint, spearmint, cherries, celery seeds, and several other plants. Its abundance makes it an easily accessible and cost-effective ingredient for fine chemical production [10]. One of its oxygenated derivatives, (R) (+)-perillyl alcohol (POH), is a stable alcohol frequently used in anti-cancer drugs. In humans, dogs, and rats, POH is rapidly metabolized into perillyl aldehyde (perillaldehyde), perillic acid, and dihydroperillic acids. These metabolites are then glucuronidated and excreted primarily in the urine, with a smaller amount being excreted in bile [11]. POH has demonstrated antitumor activity against various cancer types, including pancreatic, lung, colon, and liver cancers, in in vitro and animal studies [12].

Perillyl alcohol (POH) exhibits its anticancer properties through a combination of cellular mechanisms that translate into meaningful clinical effects. By disrupting Ras protein isoprenylation and modulating cell cycle regulators, POH inhibits tumor growth and progression, mechanisms that underpin its efficacy in reducing tumor burden. These cellular actions also correlate with its observed clinical benefits, such as extended progression-free survival in glioblastoma patients when administered intranasally. The induction of apoptosis and interference with cholesterol biosynthesis not only impair cancer cell viability but also enhance sensitivity to co-administered therapies, highlighting POH’s potential in combination treatments [11].

While its original trial with oral delivery revealed a strong dose-related gastrointestinal toxicity (including nausea and vomiting, anorexia, unpleasant taste, satiety, and eructation), its clinical translation has been reinvigorated with the development of innovative delivery methods, such as intranasal administration, which bypass systemic toxicity and effectively target glioblastomas [12]. NEO100, a highly purified POH formulation, has advanced to Phase II clinical trials, demonstrating safety and efficacy for treating recurrent glioblastomas, while early formulations faced challenges with systemic applications due to gastrointestinal toxicity. POH’s primary metabolites, perillic acid and dihydroperillic acid, as well as related compounds such as farnesol and geraniol, are currently under preclinical investigation for their enhanced therapeutic potential. Although none of these derivatives have yet received FDA approval, NEO100 is designated as an orphan drug for the treatment of glioma, and the development of these compounds reflects ongoing efforts to optimize efficacy and pharmacokinetics. These advancements highlight the importance of continued exploration of POH and its derivatives in preclinical and clinical settings, emphasizing its role as a cost-effective treatment with broad applicability in cancer therapy [12].

Although POH has already been tested on several cell types, research on other types remains limited, as the potential applications it may have. Furthermore, regarding in vitro and in vivo animal tests, it is necessary to consider some complexities in identifying the ideal dose to achieve the cellular effects necessary for a good clinical outcome. Despite the extensive evidence on the effects and mechanisms of POH in various cancer models, consolidating the currently scattered data could significantly aid in identifying new potential therapeutic applications for these limonene derivatives. Therefore, this review aims to provide a comprehensive overview of the metabolic and regulatory pathways involved in the anticancer effects of POH in different tissues, according to in vitro evidence.

## 2. Methods

### 2.1. Protocol

This review followed the PRISMA recommendations for scoping reviews (PRISMA-ScR) [13]. The PRISMA Checklist is available as Supplementary Table S1. The study protocol was registered in the Open Science Framework database, available at the link osf.io/fr2gj (accessed on 1 September 2024).

### 2.2. Sources of Information and Research Strategy

The search strategy was developed based on the PCC framework, where the following aspects were considered: population: tumor cells; concept: metabolic and regulatory pathways involved in the effects of POH; context: in vitro studies.

The search was carried out until 1 September 2024 in three different electronic databases: Pubmed, Web of Science, and Scopus. There was no limit on the date of publishing. The search key used in each database is described in Table 1. Adaptations were made to fit the same terms in different search engines and in combination with database-specific filters when available. The records were screened with the help of the online software Rayyan (https://rayyan.ai/ (accessed on 12 September 2024)) to eliminate replicas and thus consolidate the list of references for later analysis.

### 2.3. Selection of Sources of Evidence

After selecting articles from the databases, two trained and calibrated reviewers independently screened all titles and abstracts based on the eligibility criteria. These criteria included the use of tumor cell lines, in vitro exposure of POH to cells, and the investigation of regulatory and/or metabolic pathways involved in POH’s anticancer activity, such as apoptosis, cell cycle arrest, and the decreased phosphorylation of RAS/ERK pathways. Exclusion criteria included studies that did not use POH, did not involve tumor cells, did not report the metabolic or regulatory pathways of POH activity, were not in vitro studies, study designs and types of publications using other autologous materials that did not involve cellular exposure, articles that did not represent complete primary sources of evidence (abstracts, reviews, editorial letters, opinion letters, commentary articles), and in vivo or clinical studies. Furthermore, research topics unrelated to POH, such as antibacterial action, applications in diseases other than cancer, and clinical efficacy studies, were considered “off-topic”. There were no restrictions on the date or language of publication.

Two reviewers analyzed and selected the articles according to the eligibility criteria described above, eliminating duplicates. Any disagreements regarding study eligibility were resolved through discussion and consensus, with the final decision made by the senior reviewer (G.G.A) if necessary. Additionally, four other reviewers participated in the data extraction step of the selected articles. Cohen’s kappa coefficient was applied, with the two evaluators, to measure inter-examiner reliability, obtaining an index of 0.948, which is almost perfect agreement.

### 2.4. Critical Appraisal

To ensure the quality and reliability of the included studies and confirm whether they met the criteria defined for this review, a critical assessment was carried out. To this end, the ToxRTool^®^ tool was used to evaluate the inherent quality and methodological reliability of these studies.

### 2.5. Synthesis of Results and Data Charting

Data extraction was conducted by six authors during regular meetings. Key characteristics of the selected studies were collected and tabulated, including the authors and year of publication, cancer type investigated, cell type, IC_50_ values, main findings, and dose–response relationships. The extracted data were then used for a qualitative and critical analysis of the evidence.

## 3. Results

**Figure 1:**
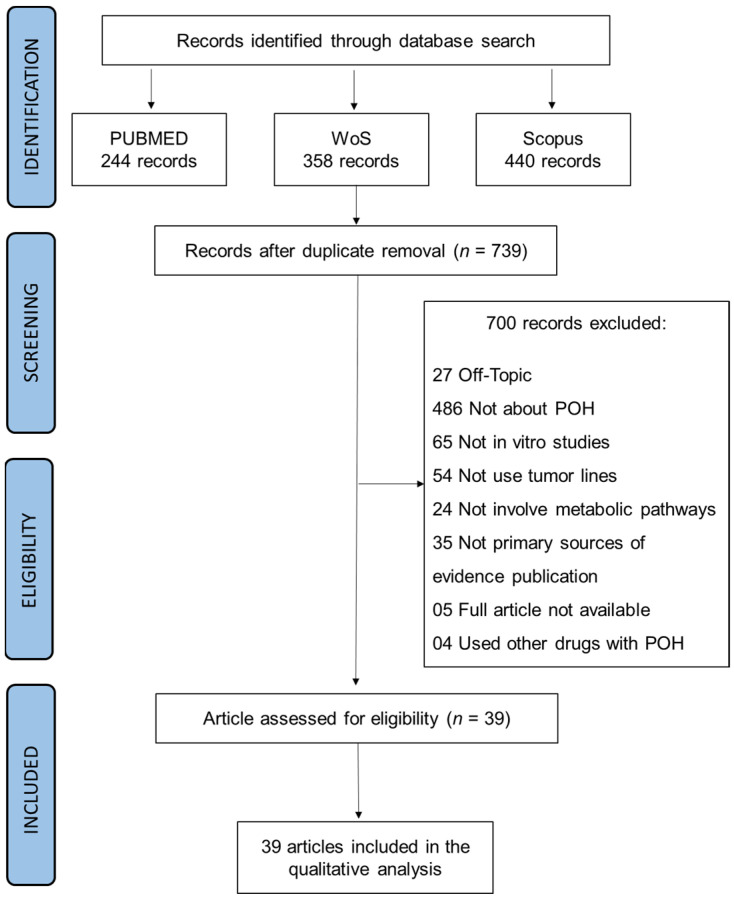
PRISMA flowchart of study screening and selection.

Figure 1 shows the results of the search strategy and screening of databases.

The PUBMED database provided 244 entries, the WoS provided 358, and Scopus provided 440. After combining the three results, 303 duplicate articles were identified and excluded. After applying the exclusion criteria, 700 articles were excluded from the review because they did not meet the eligibility criteria. From the screening result, 39 articles were selected to compose this Scoping Review.

Table 2 presents the critical evaluation performed on the selected articles at the methodological level.

Out of the 39 articles reviewed, 10 received the highest possible score (18 points), 14 articles received 17 points, 8 articles received 16 points, and 2 articles received 15 points. Although these did not achieve the maximum score, they were considered “reliable without restrictions” as they met all the essential criteria. However, one article was categorized as “not reliable”, as despite scoring 13 points, it did not score a key item related to the study design. The most common criterion that was not fulfilled across the articles was failure to report the purity of the substances (46% of the articles). Following this, the second most prevalent issue was the absence of information on the origin of the substances (26% of the articles), then articles neglecting to specify the source of cells (9%), and cases where a quantitative study was deemed infeasible (6% of the articles).

The main characteristics of the 39 selected studies related to the present research question are described in Table 3.

Notably, POH has been tested on various cancer cell lines to identify its mechanisms of action and evaluate its potential antitumor effects for different types of tumors. Only one study used primary tumor cells to conduct the study. Among the lineage tumor cells, lung cancer cells (seven articles), intestinal cancer (seven articles), glioblastoma (six articles), and breast cancer (five articles) were the most used in studies. Among the studies using cell lines derived from lung cancer, the A549 cell line was used in 63% of the trials. In studies involving glioblastoma cells, the A172 cell line was used in 50% of the cases. The HT-29 cell line was the most frequently used in intestinal cancer research, representing 43% of the trials. For breast cancer studies, the MDA-MB-231 and MCF-7 cell lines together accounted for 60% of the models chosen. Nineteen articles used different cell lines, either from the same type of cancer or different types, in the same study applying the same methodology. As expected, different dose-response and time-to-effect ratios were found. Interestingly, these differences were also present between different studies that used the same type of cell line. Furthermore, fourteen studies investigated the action of POH using animal cells. Of these, ten studies also included human cells, while four relied exclusively on animal cells as the study model. None of the selected studies compared human and animal cells to provide direct evidence of different responses to POH based on cell origin.

Although the exposure time to POH varied among different articles, most trials predominantly used a 24 h exposure period. Table 3 also shows that some studies employed different exposure times and varying doses of POH within the same study. Furthermore, it was reported that exposure to POH exerts similar effects across various tumor cell lines, including altering cell proliferation by arresting the cell cycle, inducing apoptosis, generating reactive oxygen species (ROS), decreasing endothelial growth factors, and influencing regulatory pathways involved in oncogenesis.

**Figure 2:**
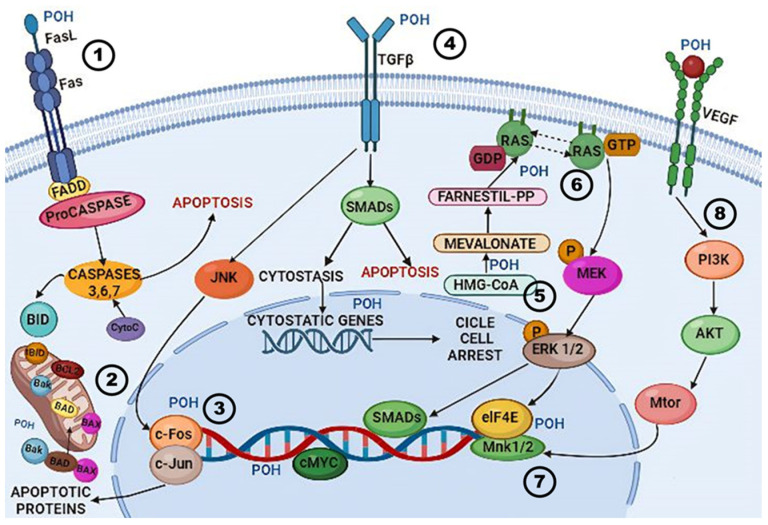
Mechanisms of action of perillyl alcohol (POH) in cancer cells, according to the in vitro evidence. POH exerts its anticancer effects through multiple mechanisms: (1) POH enhances the FasL-induced apoptosis pathway, leading to the activation of FADD and procaspase, followed by the cleavage and activation of caspases 3, 6, and 7, which activates downstream apoptotic events; (2) POH also impacts mitochondrial apoptotic pathways by regulating pro-apoptotic proteins (BAX, BAK) and anti-apoptotic proteins (BCL-2, BCL-XL); (3) POH activates the JNK pathway, leading to increased apoptosis through the activation of transcription factors such as c-Jun and c-Fos; (4) POH modulates TGF-β receptor signaling, leading to SMAD activation and the regulation of cytostatic genes, which contribute to cell cycle arrest and cytostasis; (5) POH inhibits the mevalonate pathway by blocking HMG-CoA reductase activity, thus reducing the synthesis of farnesyl-pyrophosphate and geranylgeranyl-pyrophosphate, crucial intermediates for RAS protein prenylation. This ultimately leads to inhibition of the RAS-RAF-MEK-ERK signaling cascade (6), reducing cancer cell proliferation; (7) POH inhibits eIF4E phosphorylation through Mnk1/2 signaling, contributing to the downregulation of translation of oncogenic proteins such as c-MYC, impacting cancer cell survival; (8) POH indirectly inhibits the PI3K/AKT/mTOR survival pathway, limiting cancer cell proliferation and promoting apoptosis. Created with BioRender, adapted from the content proposed by Mukhtar et al. [53].

The most investigated outcomes in the articles reviewed include the induction of apoptosis (15 articles), primarily through caspase activation (9 articles), and cell cycle arrest in the G0/G1 phases (14 articles), often due to decreased levels of cyclins and cyclin-dependent kinases (CDKs) (9 articles). Additionally, regulatory pathways frequently altered in various cancers were examined, such as increased expression of the cyclin inhibitor p21 (six articles) and decreased activation of the AKT pathway (five articles), leading to reduced cell proliferation and survival. Other fundamental but less investigated pathways include the inhibition of protein isoprenylation and decreased phosphorylation of the extracellular signal-regulated kinase (ERK), MEK (mitogen-activated protein kinase), and mitogen-activated protein kinase (MAPK) pathways. The effects of POH on the activity of PARP-1 (Poly (ADP-ribose) polymerase 1), Na/K-ATPase, Fas ligand, and the RAS pathway were also investigated. These results are summarized in Figure 2.

The half maximal inhibitory concentration (IC_50_) is a measure used to assess the effectiveness of a substance in inhibiting a specific biological function or activity by 50%. This is one of the most crucial tools in drug development as it helps to evaluate the effectiveness of drugs, guide dosing strategies mainly to animal tests, and understand the mechanisms of action of inhibitors and their potential therapeutic applications. Despite its significance, as shown in Table 3, only 19 out of the 39 articles included in this review investigated the IC_50_ value of POH.

According to the analyzed data, MCF-7 cells exhibited the lowest IC_50_ values compared to other cell lines, indicating they were the most sensitive to POH. An important fact to note is that studies using the same cell line, such as A549, reported varying IC_50_ values. This variability indicates that IC_50_ values can be significantly influenced by different experimental conditions.

Another essential tool in studies of new molecules with potential antitumor properties is the calculation of the selectivity index (SI), which measures how effectively a molecule kills tumor cells compared to normal cells. It is essential for determining whether to continue studying a particular drug. Out of 39 articles, only 9 investigated cytotoxicity or regulatory pathways using normal cells, and only Oturanel et al. [39] calculated the SI. Of the nine articles that used normal cells, only two articles exclusively used human cells for comparison, three articles used both normal human and animal cells, while four articles used only murine cells, including the one that calculated the SI value.

## 4. Discussion

Cancer is a global health challenge, responsible for over 10 million deaths annually [1]. Chemotherapeutic agents, which act systemically, are one of the primary treatments for cancer [54]. However, their narrow therapeutic window and the development of tumor resistance mechanisms complicate the management of side effects, necessitating the continuous search for new therapeutic approaches [55].

POH, a plant-derived compound, has shown significant antineoplastic effects by modulating various cellular pathways. In this review, POH demonstrated a wide range of actions on tumor cells, impacting pathways related to proliferation, cell cycle regulation, resistance mechanisms, DNA synthesis, and cholesterol synthesis. These effects culminated in apoptosis, cell cycle arrest, and the inhibition of migration and tumor invasiveness, as observed in several analyzed articles.

### 4.1. Elucidating the Regulatory Pathways Underpinning the Biological Effects of Perillyl Alcohol

#### 4.1.1. Cell Cycle Regulation

The cell cycle is a highly coordinated process that involves a series of events leading to cell division and proliferation. During the cell cycle, cyclins are synthesized and degraded in a timely manner to activate specific CDKs at different checkpoints. In the G1 phase, cyclin D pairs with CDK4/6 to regulate progression towards DNA synthesis in the S phase [56]. Cyclin E then interacts with CDK2 to initiate DNA replication and entry into the S phase. Subsequently, cyclin A associates with CDK2 to facilitate DNA synthesis and progression through the S phase [56]. As cells prepare for mitosis, cyclin A is degraded, and cyclin B binds to CDK1 to orchestrate mitotic entry in the M phase [56]. The timely degradation of cyclins ensures proper checkpoint control and prevents aberrant cell cycle progression. In cancer, cyclins are often overexpressed, which leads to the uncontrolled activation of CDKs, which drive the cell cycle forward without the usual regulatory checks, allowing cancer cells to continue dividing uncontrollably, even in the presence of DNA damage or other abnormalities [57].

POH has been shown to modulate the levels of specific cyclins, which in turn affects CDK activity and influences cell cycle progression. This modulation can alter cell cycle checkpoints, potentially leading to either cell cycle arrest or progression through various phases. Shi et al. [46] found that POH induces G0/G1 cell cycle arrest in murine-transformed mammary epithelial cells, likely due to decreased activity of cyclin D1, cyclin A, cyclin D1-associated kinase, and cyclin E-CDK2. Similarly, Yuri et al. [52] and Bardon et al. [14] reported that POH causes G0/G1 cell cycle arrest in human breast cancer cells by reducing cyclin D1 and cyclin E levels. In contrast, in another study by Bardon et al. [15], it was observed that in human colorectal cancer, POH reduced cyclin D1, cyclin-dependent kinases CDK4, and CDK2 but resulted in an increase in cyclin E. Rajesh and Howard [42] reported an increase in the G2/M phase of the cell cycle and elevated cyclin D1 expression in human prostate carcinoma following POH treatment. Wiseman et al. [49] found that POH led to G0/G1 cell cycle arrest, increased cyclin D1 levels, and decreased cyclin A, cyclin B1, and CDK2 activity in human pancreatic adenocarcinoma.

The observed increase in cyclin D1 and the G2/M phase can be attributed to the complex interactions between anticancer drugs and cellular mechanisms. Anticancer agents that cause DNA damage can activate cell cycle checkpoints, leading to increased cyclin D1 as cells attempt to repair the damage and continue through the cycle [58]. Additionally, cancer cells may upregulate cyclin D1 as a compensatory mechanism to counteract drug effects and maintain proliferation, leading to drug resistance [59,60]. Thus, the impact of POH on the cell cycle, through its regulation of cyclins, highlights its potential as a modulator of cellular proliferation and a promising therapeutic target in cancer treatment.

p21Waf1/Cip1, also known as cyclin-dependent kinase inhibitor 1 (CDKN1A) or simply p21, is a crucial protein involved in the regulation of the cell cycle and cell proliferation [61]. It functions primarily by inhibiting cyclin-CDK complexes, leading to cell cycle arrest, particularly at the G1 checkpoint [61]. Regulated by the tumor suppressor p53 [62], p21 plays a critical role in halting cell cycle progression and inducing cell cycle arrest in response to cellular stresses, such as DNA damage or oncogene activation [63]. Additionally, p21 contributes to apoptosis by modulating pro- and anti-apoptotic proteins, thereby aiding in the removal of damaged or unwanted cells [64]. Dysregulation of p21 is common in various cancers, enabling tumor cells to bypass cell cycle checkpoints and proliferate uncontrollably. This dysregulation underscores the potential of targeting p21 as a therapeutic strategy in cancer treatment [65].

POH has been shown to increase p21 expression across several cancer models, including human colon cancer [15,34], murine-transformed mammary epithelial cells [46], human pancreatic adenocarcinoma [49], human breast cancer [52], and human lung adenocarcinoma and squamous cell carcinoma [51].

#### 4.1.2. Apoptosis

The caspase cascade is a precisely regulated mechanism crucial for the process of apoptosis. Caspases are typically present in cells as inactive precursors, known as procaspases [66]. In response to apoptotic signals, these procaspases are cleaved to form active caspases, which then perform apoptosis by degrading key cellular components [66]. Caspases are categorized into initiator caspases, such as caspase-8, which are involved in the early stages of apoptosis and are responsible for activating downstream effector caspases [67], and into effector caspases, such as caspase-3 and caspase-7, which play a central role in the final stages of apoptosis by cleaving various cellular substrates [67]. Caspase-3 is one of the most critical caspases involved in the execution phase of cell death. Once activated, caspase-3 cleaves various cellular substrates, like poly (ADP-ribose) polymerase (PARP) and caspase-activated DNase (CAD), leading to the hallmark features of apoptosis, such as cell shrinkage, chromatin condensation, nuclear fragmentation, and membrane blebbing [68]. Dysregulation of caspase-3 is commonly observed in cancer, where reduced activity can contribute to apoptosis resistance, allowing cancer cells to evade death and proliferate uncontrollably [67].

Some articles included in this review have demonstrated that POH induces cell death by acting on the late stages of apoptosis, increasing the activity of caspase-3 [25,35,39,50,51] and caspase-7 [37]. For instance, Rajesh et al. [43] reported that POH enhances early apoptotic events by upregulating caspase-8 activity.

Poly (ADP-Ribose) Polymerase 1 (PARP-1) is a key enzyme involved in DNA repair, especially in the repair of single-strand DNA breaks [69]. Upon sensing DNA damage, PARP-1 initiates the repair process by adding ADP-ribose polymers to nuclear proteins, facilitating the recruitment of repair factors [70]. However, PARP-1 also plays a role in apoptosis. During this process, caspase-mediated cleavage of PARP-1 leads to its inactivation, halting DNA repair and promoting caspase-driven DNA fragmentation, a hallmark of apoptotic cell death [71]. In studies investigating the effects of POH on PARP-1 regulation, de Lima et al. [25] and Xu et al. [50] found that POH increased the cleavage of PARP-1, decreasing its activity in human lung cancer cell lines. Unlike Marin-Ramos et al. [37], increased PARP-1 activity in primary human glioblastoma cells following POH treatment was observed. Elevated PARP-1 activity is implicated in several mechanisms that contribute to tumor cell resistance to therapy. As a key DNA repair enzyme, PARP-1 helps maintain genomic stability in tumor cells after treatment with DNA-damaging chemotherapeutic agents, thereby enhancing their survival [72].

FAS ligand (FASL or CD95L) is a type II transmembrane protein of the tumor necrosis factor (TNF) family [73,74]. It serves as the ligand for the FAS receptor (FAS or CD95) [74]. The binding of FASL to FAS triggers the formation of the death-inducing signaling complex (DISC), leading to apoptosis through the activation of caspases, particularly caspase-8 [74]. Rajesh et al. [42,43] reported an increase in the expression of FAS and the membrane-bound form of Fas-L after exposing human prostate carcinoma, human glioblastoma, human astrocytoma, and rat glioma cells to POH.

The FASL-FAS interaction can intersect with the AKT pathway, as AKT activation promotes cell survival and can counteract apoptotic signals [75]. The AKT pathway modulates the sensitivity of cells to FASL-induced apoptosis, and in cancer, this pathway is often dysregulated, leading to enhanced cell survival and resistance to apoptosis [76].

#### 4.1.3. AKT Pathway

The AKT pathway, also known as the PI3K/AKT/mTOR pathway, is a critical signaling cascade that regulates a wide range of cellular processes, including growth, survival, metabolism, and proliferation [77]. Activation of PI3K (phosphoinositide 3-kinase) occurs in response to various signals, such as growth factors binding to receptor tyrosine kinases (RTKs) [78]. Once activated, PI3K phosphorylates phosphatidylinositol 4,5-bisphosphate (PIP2) in the cell membrane, generating phosphatidylinositol 3,4,5-trisphosphate (PIP3). PIP3 acts as a docking site for AKT, recruiting it to the cell membrane [78]. At the membrane, AKT undergoes full activation through phosphorylation by PDK1 (3-phosphoinositide-dependent kinase-1) and mTORC2 (mammalian target of rapamycin complex 2) [78]. This pathway is tightly regulated by several mechanisms, including the tumor suppressor PTEN (phosphatase and tensin homolog), which dephosphorylates PIP3 back to PIP2, thereby inhibiting AKT signaling [79].

Once activated, AKT detaches from the membrane and translocates to the cytoplasm and nucleus, where it phosphorylates numerous substrates involved in various cellular functions. AKT promotes cell survival by inhibiting pro-apoptotic factors, such as the BAD [80], and by activating anti-apoptotic proteins like the Bcl-2 family [81]. It also stimulates cell growth by enhancing protein synthesis through mTORC1 activation and by regulating the cell cycle. This pathway is frequently dysregulated in cancer due to mutations in its components, such as PI3K and PTEN, or in upstream activators like RTKs [80]. Hyperactivation of the AKT pathway results in uncontrolled cell growth, enhanced survival, and resistance to apoptosis, all of which contribute to tumorigenesis and drug resistance [78].

Loutrari et al. [35] found that POH decreased AKT phosphorylation in bovine lung microvascular endothelial cells (BLMVEC), but no significant effect was observed in the AKT pathway in leukemia K562 cells. Peffley et al. [41] reported a decrease in AKT phosphorylation in DU145 but no difference in PC3 cells. In contrast, Marin-Ramos et al. [37] reported that POH reduced AKT phosphorylation in primary glioblastoma cells. Interestingly, Ma et al. [36] observed an increase in AKT phosphorylation in HCT116, SK-Hep1, and HeLa cells. This increase may be explained by the decreased phosphorylation of mTOR observed in their experiment. Reduced mTOR activity can disrupt feedback loops within the PI3K/AKT/mTOR pathway. For example, when mTORC1 activity is reduced, it can relieve negative feedback inhibition on upstream components like PI3K, potentially leading to hyperactivation of AKT if other regulatory mechanisms are not engaged [82].

#### 4.1.4. RAS/RAF/MEK/ERK Pathway

RAS is a small GTPase that switches between an active, GTP-bound state and an inactive, GDP-bound state [83]. The primary isoforms of RAS include KRAS, HRAS, and NRAS [84]. When stimulated by upstream signals, such as the binding of growth factors to receptor tyrosine kinases (RTKs), RAS activates the RAF kinases [85]. RAF, a family of serine/threonine-specific protein kinases, then phosphorylates and activates MEK upon activation by RAS [85]. Subsequently, MEK1 and MEK2, which are dual-specificity kinases, phosphorylate and activate extracellular signal-regulated kinase (ERK) [84,85]. In cancer, mutations in RAS result in the persistent activation of RAS proteins, leading to continuous stimulation of the RAS/RAF/MEK/ERK pathway, which drives uncontrolled cell proliferation and tumor development [84].

The duration and intensity of ERK signaling are critical determinants of cellular outcomes [86,87]. Sustained moderate ERK activation over several hours can downregulate anti-proliferative genes, thereby promoting the transition of cells from the G0/G1 phase into the S phase of the cell cycle [88]. This process is essential for the expression of pro-proliferative signals, such as cyclin D1. In contrast, transient but higher levels of ERK activity can trigger the expression of CDK inhibitors like p21 and p27, leading to cell cycle arrest [87].

The RAS–RAF–MEK–ERK pathway is intricately connected to the RAS–PI3K–AKT–mTOR pathway, with both pathways capable of influencing each other at various points [83]. For example, AKT can phosphorylate and inhibit RAF (a component of the RAS pathway), thereby modulating ERK signaling [89,90]. Conversely, activation of the ERK pathway can suppress PI3K signaling through different feedback mechanisms. When one pathway is inhibited, the other may be upregulated as a compensatory response [91]. This interplay is frequently observed in cancer cells, where blocking the PI3K pathway can lead to increased activity in the ERK pathway and vice versa [92,93].

Research into the effects of POH on this pathway has yielded varied results. Koyama et al. [35] observed a reduction in ERK phosphorylation in K562 leukemia cells following POH treatment. Silva et al. [47] had the same result with ONS76 and DAOY cells, but with UW402 and UW473, the phosphorylation increased. Clark et al. [22] found a reduction in ERK and MEK in K562 and Bcr/Abl-transformed FDC-P1 and 32D. Similarly, Marin-Ramos et al. [37] reported a decrease in p42/44 MAPK phosphorylation in human glioblastoma primary cells. In contrast, Karlson et al. [33] found no effect of POH on MAPK activity in PANC-1 human pancreas carcinoma cells. Fischer et al. [29] noted an increase in RAS protein levels but a decrease in ERK phosphorylation in A172 human glioblastoma cells. This paradoxical result could be explained by several factors. The inhibition of components like MEK or ERK may trigger feedback loops that enhance the expression or stability of upstream proteins such as RAS [94]. Furthermore, certain drugs may selectively inhibit ERK phosphorylation without altering RAS protein levels. For instance, MEK inhibitors block the activation of ERK by targeting MEK directly [95,96]. Also, cancer cells often deploy compensatory mechanisms that elevate RAS protein levels in response to downstream pathway inhibition, attempting to sustain proliferative signals despite drug treatment [97,98].

Da Gama Fisher et al. [28] also observed an increase in p42 phosphorylation but a decrease in p44 phosphorylation in human lung carcinoma cells (A549). These results might be due to selective inhibition, where certain drugs selectively inhibit the phosphorylation of p44 without affecting or even enhancing the phosphorylation of p42 [96,99]. Feedback mechanisms could also play a role, where inhibiting one protein in the pathway prompts compensatory responses that alter the phosphorylation status of other proteins [100,101]. Additionally, p42 and p44, though closely related, can be differentially regulated by upstream kinases and phosphatases [102,103].

#### 4.1.5. Isoprenylation of RAS Proteins

Isoprenylation is a vital post-translational modification that plays a critical role in the functionality of the RAS signaling pathway. This modification involves the covalent attachment of isoprenoid groups, such as farnesyl or geranylgeranyl, to the cysteine residue located at the C-terminal of RAS proteins [104,105]. This process is catalyzed by the enzymes farnesyltransferase (FTase) and geranylgeranyltransferase (GGTase) [104,105]. Isoprenylation facilitates the anchoring of RAS proteins to the plasma membrane, a localization that is essential for their interaction with upstream activators and downstream effectors [106]. Without isoprenylation, RAS proteins fail to properly localize to the membrane, significantly impairing their ability to transmit signals that regulate cell growth, differentiation, and survival [105,106]. The process of isoprenylation is tightly regulated by the availability of isoprenoid precursors, which are synthesized via the mevalonate pathway [107]. This pathway is a key metabolic route targeted by statins, drugs that inhibit HMG-CoA reductase [108,109].

In the context of cancer, the isoprenylation of RAS proteins is crucial for the oncogenic activity of mutant RAS, which is frequently found in various malignancies [110]. The proper membrane localization and function of RAS are essential for the sustained proliferative signaling that drives tumor development and progression. Targeting the isoprenylation process has thus emerged as a potential therapeutic strategy in cancers driven by aberrant RAS signaling [106,110].

Crowell et al. [24] reported a decrease in the isoprenylation of small G proteins in human colon adenocarcinoma cells (HT-29) following exposure to POH. Clark et al. [22] verified that POH did not inhibit protein prenylation in Bcr/Abl-transformed FDC-P1 and 32D. Similar findings were observed by Cerda et al. [18] in human colonic cancer cells (SW480) and in murine colon cancer cells (CT-26). Interestingly, despite the inhibition of isoprenylation, these studies also noted an increase in cholesterol synthesis. This paradoxical result may be due to the inhibition of isoprenylation, prompting the cells to enhance the production of isoprenoid intermediates as a compensatory response [111,112]. This upregulation likely extends to the entire mevalonate pathway, leading to increased cholesterol synthesis as part of the cellular attempt to restore balance in the pathway [112,113].

#### 4.1.6. Na/K-ATPase

The Na/K-ATPase, commonly known as the sodium-potassium pump, is a crucial enzyme located in the plasma membrane of cells [114]. It primarily functions to maintain the electrochemical gradients of sodium (Na⁺) and potassium (K⁺) ions across the cell membrane through active transport, which is essential for numerous physiological processes [114]. By sustaining these ion gradients, the Na/K-ATPase regulates cell volume and osmotic balance [115], which are critical for cell division and proliferation [116]. The Na/K-ATPase also functions as a receptor coupled with the Src family of non-receptor tyrosine kinases, forming a functional complex for signal transduction [117]. For cell–cell-induced effects, the SRC/RAS/RAF/MEK/ERK pathway is implicated, while ion current enhancement involves the PI3K/AKT/mTOR pathway [118].

Moreover, the Na/K-ATPase interacts with various signaling pathways, including the Src family kinases and the PI3K/Akt pathway, thereby influencing cell growth and proliferation [119]. The proper functioning of this pump is also essential for cell motility, as it modulates the actin cytoskeleton and focal adhesions, which are crucial for cell movement and invasion [120]. Disordering in these pathways can facilitate cancer cell detachment and metastasis [121].

The Na/K-ATPase also regulates intracellular calcium levels, influencing the activity of the Na+/Ca^2+^ exchanger (NCX), which is important for apoptotic signaling [118,121]. Imbalances in calcium homeostasis can inhibit apoptosis, allowing cancer cells to survive and proliferate. Additionally, changes in the activity of this pump can affect metabolic pathways that provide energy and biosynthetic materials for rapidly growing cancer cells [121,122]. It has been found that POH can reduce Na/K-ATPase activity in human glioblastoma cells [30,31].

### 4.2. Methodological Implications

Cell selection plays a pivotal role in the design and execution of in vitro drug studies. The choice of cell line or primary cells can significantly influence the relevance, reproducibility, and translatability of the results [123]. Studies have shown that tumor cell lines established decades ago do not adequately reproduce the drug sensitivity and behavior of the original cancers from which they were derived [124]. Notably, only one article in this review used primary tumor cells to investigate regulatory pathways of POH.

Another key consideration when choosing a cell type is that while the metabolism of animal and human cells shares many fundamental biochemical pathways due to their common evolutionary ancestry and cellular machinery, there are also critical differences. These differences can influence experimental outcomes and pose challenges in translating findings to human health applications. Species-specific genes can lead to distinct metabolic pathways, enzyme activities, and epigenetic modifications, such as DNA methylation and histone acetylation, which influence gene expression and cellular metabolism [125]. Enzyme activity and expression can vary substantially between species, affecting drug metabolism. For example, cytochrome P450 enzymes function differently across species [126]. Rodents, for instance, often metabolize certain drugs more rapidly than humans, which can alter drug efficacy and toxicity profiles [127,128]. For these reasons, human cell lines or primary cells are often preferred over animal cells, as they provide more accurate predictions of human physiological responses and clinical outcomes.

When selecting cell lines for research, it is essential to verify that the cell lines have not been misidentified or cross-contaminated. Although exact figures are not readily available, estimates suggest that approximately 20% of cell lines are incorrectly labeled, often due to intraspecies contamination [129,130]. For example, the KLP-1 cell line used in one study of this review was found to be contaminated with MCF-7 cells [131]. HeLa cells, known for their high chromosomal instability, have been shown to exhibit genomic variability between different batches, which can lead to differences in gene expression and result in inconsistent experimental outcomes [132].

Several articles in this review reported obtaining cells from other researchers, a practice that can exacerbate problems related to non-traceability. Ensuring proper traceability is crucial, as factors such as high passage numbers, extended generation times, and mycoplasma contamination can adversely affect experimental results [133]. Notably, none of the articles reviewed reported the passage number of the cells used. All these cell-related factors could contribute to variations in results across studies using the same cell line, highlighting the importance of careful cell selection and authentication in in vitro drug research.

Calculating IC_50_ is also a fundamental aspect of anticancer drug testing in vitro, providing essential information about drug potency, efficacy, dose–response relationships, resistance mechanisms, and overall drug development [134]. This metric is vital for the selection and optimization of therapeutic agents, ensuring that the most promising candidates advance to clinical testing [134]. This quantitative analysis allows researchers to assess how different concentrations of a compound affect the regulatory pathway in question.

Moreover, the determination of the SI is a critical aspect of anticancer drug discovery. The SI ensures that new treatments are effective against cancer cells while minimizing harm to normal cells, thereby optimizing therapeutic efficacy and improving cost-effectiveness and patient quality of life [135]. In the reviewed literature, less than half of the selected articles determined the IC_50_ and only one article determined the SI value. Furthermore, most articles did not provide justification for the tested POH doses, as they were not based on IC_50_ values. This points to the need for more rigorous determination and reporting of IC_50_ and SI values in anticancer drug research to ensure the validity and applicability of the findings, considering that unspecific and non-selective toxicity may be one of the main drawbacks of the therapeutical use of POH and its derivatives.

### 4.3. Limitations and Summary of Evidence

The present scoping review has certain limitations that affect the scope and depth of the analysis. A significant limitation is the exclusive focus on peer-reviewed articles, which excludes potentially valuable insights from grey literature. Additionally, focusing on studies investigating metabolic pathways may have excluded relevant quantitative evidence on the selectivity index. Despite these limitations, the review successfully delineates the regulatory pathways through which POH exerts its biological effects in cancer.

This literature review provides a comprehensive overview of studies exploring the mechanisms of action of POH in various tumor cell lines, identifying foundational work conducted during the last thirty years. POH modulates specific cyclin levels and upregulates p21, influencing CDK activity and consequently affecting cell cycle progression. Furthermore, POH suppresses cell growth and promotes cell death by attenuating AKT phosphorylation and enhancing PARP-1 activity across various cell types. It also induces apoptosis by increasing caspase activity and modulating FAS and FASL sensitivity. Additionally, POH reduces ERK phosphorylation, inhibits RAS protein isoprenylation, and decreases Na/K-ATPase activity, demonstrating its multifaceted impact on cellular behavior and signaling pathways. Understanding these regulatory pathways is essential for uncovering new therapeutic applications and potential opportunities for drug repositioning, ultimately advancing the development of novel cancer treatments.

## 5. Conclusions

This systematic review highlights the broad anticancer potential of perillyl alcohol (POH) across various tumor cell lines and its multifaceted actions on cellular pathways, including cell cycle regulation, apoptosis, and inhibition of oncogenic signaling cascades. While POH demonstrates significant promise as a therapeutic agent, several critical gaps remain in the current literature that warrant further investigation to fully realize its clinical applicability. First, the lack of studies on less commonly investigated cancer types, such as osteosarcoma and other solid tumors, limits our understanding of POH’s broader therapeutic potential. Second, while traditional in vitro methodologies (e.g., PCR, FACS, Western blot, and ELISA) dominate the field, modern approaches like 3D cell cultures, microfluidics, omics technologies, and adverse outcome pathway (AOP) frameworks have not been widely integrated into POH research. These advanced methodologies could provide a more physiologically relevant context and yield insights into the compound’s mechanisms and therapeutic windows. Additionally, the selectivity of POH’s effects remains insufficiently explored. Most studies reviewed did not employ normal cells or calculate the selectivity index (SI), hindering our ability to address concerns regarding toxicity and therapeutic efficacy. Improved selectivity analysis could aid in overcoming challenges associated with POH’s safety profile and facilitate its approval for clinical applications. Finally, while the clinical investigation of inhaled POH formulations, such as NEO100 for glioblastoma, has progressed, a comprehensive exploration of optimized delivery systems and combinatorial regimens with other therapeutic agents remains largely absent. Future research should prioritize these directions to build on the foundational knowledge and drive the translation of POH into effective and accessible cancer therapies.

## Figures and Tables

**Table 1:** Search key utilized in the three consulted databases.

Database	Search Key
PubMed (https://pubmed.ncbi.nlm.nih.gov/ (accessed on 1 September 2024))	(Anticancer activity OR Chemotherapeutic agents OR Tumor regression OR Anticancer agent OR Antineoplastic Agents [mh] OR chemotherapy *) AND (perillyl alcohol [mh] OR Perillic acid [mh] OR POH OR Limonene metabolites OR Limonene derivative OR limonene [mh]) AND (in vitro OR Cell culture OR Tumor cells OR in vitro techniques [mh])
Web of Science (https://www.webofscience.com/ (accessed on 1 September 2024))	((Anticancer activity OR Chemotherapeutic agents OR Tumor regression OR Anticancer agent OR Antineoplastic Agents OR chemotherapy *) AND (perillyl alcohol OR Perillic acid OR POH OR Limonene metabolites OR Limonene derivative OR limonene) AND (in vitro OR Cell culture OR Tumor cells OR in vitro techniques))
Scopus (https://www.scopus.com/search/form.uri?display=basic (accessed on 1 September 2024))	TITLE-ABS-KEY (“Anticancer activity” OR “Chemotherapeutic agents” OR “Tumor regression” OR “Anticancer agent” OR “Antineoplastic Agents” OR chemotherapy OR anticancer) AND TITLE-ABS-KEY (“perillyl alcohol” OR “Perillic acid” OR POH OR “Limonene metabolites” OR “Limonene derivative” OR limonene) AND TITLE-ABS-KEY (“in vitro” OR “Cell culture” OR “Tumor cells” OR “in vitro techniques”)

**Table 2:** Result of the critical appraisal of the included articles according to ToxRTool^®^.

Study	Group I: Substance Identification	Group II: Test System Characterization	Group III: Study Design Description	Group IV: Study Result Documentation	Group V: Plausibility of Study Design	Total	Reliability Categorization
Bardon et al. [14]	3	3	6	3	1	16	Reliable without restrictions
Bardon et al. [15]	3	3	4	3	0	13	Not reliable
Berchtold et al. [16]	3	3	6	3	2	17	Reliable without restrictions
Broitman et al. [17]	2	3	6	3	2	16	Reliable without restrictions
Cerda et al. [18]	3	3	6	3	2	17	Reliable without restrictions
Chan et al. [19]	3	3	6	2	1	15	Reliable without restrictions
Chung et al. [20]	3	3	6	3	2	17	Reliable without restrictions
Clark et al. [21]	3	2	6	3	2	16	Reliable without restrictions
Clark et al. [22]	3	3	6	3	2	16	Reliable without restrictions
Clark [23]	3	3	5	3	2	16	Reliable without restrictions
Crowell et al. [24]	4	3	6	3	2	18	Reliable without restrictions
de Lima et al. [25]	4	3	6	3	2	18	Reliable without restrictions
de Saldanha et al. [26]	2	3	6	3	2	16	Reliable without restrictions
Elegbede et al. [27]	3	3	6	3	2	17	Reliable without restrictions
da Gama Fisher et al. [28]	4	3	6	3	2	18	Reliable without restrictions
Fischer et al. [29]	4	3	6	3	2	18	Reliable without restrictions
Garcia et al. [30]	4	3	6	3	2	18	Reliable without restrictions
Garcia et al. [31]	3	3	6	3	2	17	Reliable without restrictions
Gehauser et al. [32]	4	3	6	3	2	18	Reliable without restrictions
Karlson et al. [33]	4	3	5	3	2	17	Reliable without restrictions
Koyama et al. [34]	3	3	6	3	2	17	Reliable without restrictions
Loutrari et al. [35]	3	3	6	3	2	17	Reliable without restrictions
Ma et al. [36]	4	3	6	3	2	18	Reliable without restrictions
Marín-Ramos et al. [37]	3	3	6	3	2	17	Reliable without restrictions
Nandurkar et al. [38]	3	2	6	3	2	16	Reliable without restrictions
Oturanel et al. [39]	2	3	6	3	2	16	Reliable without restrictions
Paduch et al. [40]	4	3	6	2	1	16	Reliable without restrictions
Peffley et al. [41]	3	3	6	3	2	16	Reliable without restrictions
Rajesh and Howard [42]	3	3	6	3	2	17	Reliable without restrictions
Rajesh et al. [43]	3	3	6	3	2	17	Reliable without restrictions
Sahin et al. [44]	3	3	6	3	2	16	Reliable without restrictions
Shi and Gould [45]	4	3	6	3	2	18	Reliable without restrictions
Shi and Gould [46]	4	3	6	3	2	18	Reliable without restrictions
Silva et al. [47]	3	3	6	3	2	16	Reliable without restrictions
Stayrook et al. [48]	3	3	6	3	2	17	Reliable without restrictions
Wiseman et al. [49]	4	3	6	3	2	18	Reliable without restrictions
Xu et al. [50]	1	3	6	3	2	15	Reliable without restrictions
Yeruva et al. [51]	3	3	6	3	2	17	Reliable without restrictions
Yuri et al. [52]	4	2	6	3	2	17	Reliable without restrictions

**Table 3:** Main results of the included articles.

References	Cancer Type	Normal Cell	Tumor/Transformed Cell	Exposure Time	Reported Dose	Results After POH Exposure	IC_50_
Bardon et al. [14]	Human breast cancer	-	T-47D	6 days	10 μM	Cell growth (−)	T-47D = 0.1 mM
				48 h	0.5 mM	Cell cycle arrest in G1	
				24 h		Cyclin D1 mRna (−)	
			MCF-7	6 days	0.1 mM	Cell growth (−)	
				24 h	0.5 mM	Cyclin D1 mRna (−)	
			MDA-MB-231	6 days	0.1 mM	Cell growth (−)	
				24 h	1 mM	Cyclin D1 mRna (−)	
Berchtold et al. [16]	Human breast cancer	-	MDA-MB-468	24 and 48 h	Not informed	NF-κB DNA-binding (−), calcium-dependent NF-κB activity in an estrogen receptor-independent (ER) (−)	-
	Human American Burkitt’s lymphoma		Ramos cells	24 h	0.7 mM	Apoptosis (+)	
	Murine lymphoma		WEHI-231	24 h	0.7 mM	Apoptosis (+), NF-κB DNA-binding (−), IκBα mRNA expression (−), Bcl-2 gene family, and Bfl-1/A-1 (−)	
				30 and 60 min	0.7 mM	Calcium concentrations (−)	
Chan et al. [19]	Human breast cancer	-	MCF-7	24 h	0.5 μM	EROD (−), CYP1B1 (−), XRE-driven Luciferase activities (−)	1.5 μM
Yuri et al. [52]	Human breast cancer	-	KPL-1, MCF-7, MDA-MB-231 and MKL-F (transfectant of MCF-7)	48 and 72 h	500 μM	All cells: G0/G1 arrest (+), cell proliferation (−); KPL-1 and MLK-F: p21 protein (+), cyclin D1 and cyclin E (−)	-
Loutrari et al. [35]	Human breast cancer		MDA-MB-231	24 h	0.2 mM to 1 mM	Production of VEGF (−)	-
	Mouse melanoma		B16	24 h	0.2 mM to 1 mM	Production of VEGF (−)	
	-	HUVEC	-	6 days	0.5 mM	Differentiation of endothelial cells into tube-like structures (−)	
		BLMVEC	-				
				48 h	1 mM	Endothelial cell growth (−), caspase-3 (+)	
				Not informed	Not informed	Ang2 release (+)	
				15 to 120 min	0.5 mM to 1 mM	Akt phosphorylation (−)	
	Human myelogenous leukemia		K562	72 h	0.1 mM to 1 mM	Cell growth (−), caspase-3 (+), apoptosis (+), did not alter Akt phosphorylation, ERK1/2 phosphorylation (−), production of VEGF (−)	
Clark [23]	Human myelogenous leukemia	-	Bcr/Abl-transformed FDC-P1 and 32D, and K562	8 h	400 μM to 1 mM	Did not affect the level of c-Myc RNA	Bcr/Abl-transformed FDC-P1 = 400 μM
				24 h	500 μM (normal cell) and 1 mM (K562)	Cell growth (−), apoptosis (+)	
Clark et al. [22]	Ph^+^ leukemias	-	Bcr/Abl-transformed FDC-P1 and 32D, and K562	24 h	400 μM	FDC-P1: DNA synthesis (−), G0/G1 arrest (+), apoptosis (+)	-
				8 h	200 μM to 1 mM	ERK and MEK activity (−), did not affect the kinase activity of Bcr/Abl, did not inhibit protein prenylation or Ras activation	-
Clark et al. [21]	Ph^+^ leukemias	-	Bcr/Abl-transformed FDC-P1 and 32D	16 h	300 μM and 600 μM	Apoptosis (+), cell viability (−), cell cycle arrest in G0/G1 (+)	-
Gerhäuser et al. [32]	Human promyelocytic leukemia	-	HL-60	4 days	>100 μM	Did not affect radical scavenging	HL-60 > 100 μM
	Abelson murine leukemia virus-induced tumor	-	Raw 264.7	24 h	>50 μM	Inhibition of iNOS induction (+)	
	Papilloma of the mouse skin	-	Mouse 308	Not informed	>10 μM	No influence in ornithine decarboxylase (ODC) inhibition	
	Rat hepatoma cells	-	H4IIE	38 h	>50 μM	No influence in Cyp1A inhibition and QR induction	
Sahin et al. [44]	Ph^+^ leukemias	FDC-P1 and 32D	Bcr/Abl-transformed FDC-P1 and 32D	16 h	500 μM	Inhibits growth of Bcr/Abl-transformed cells (+), G0/G1 arrest (+), apoptosis (+)	FDC.P1 = 900 μM; transformed 32D = 300–400 μM; transformed FDC.P1 = 400 μM
Bardon et al. [15]	Human colorectal cancer	-	HCT116	6 days	0.25 mM	Cell growth (−)	0.5 mM
				48 h	0.75 mM and 1 mM	Cell cycle arrest in G0/G1	
				24 h	0.25 mM to 1 mM	p21 (+), cyclin E (+), cyclin D1 (−), CDK4 and CDK2 (−)	
Broitman et al. [17]	Murine colon cancer	-	CT-26	48 h	1 mM	Cholesterol synthesis (+)	-
				24 h	1 mM	Isoprenylation (+)	
				1 to 4 days	0.5 mM and 1 mM	No effect on cell growth	
Cerda et al. [18]	Human colonic cancer	-	SW480	24 h	1 mM	Cholesterol synthesis (+), protein isoprenylation (−), LDL internalization (−), did not affect microsomal HMG-CoA reductase activity	-
Crowell et al. [24]	Human colon adenocarcinoma	NIH3T3, MCF-10 and M600B	HT-29	10 h	1 mM	Cell proliferation (−), protein isoprenylation (−)	HT29 = 50 μM
Koyama et al. [34]	Human colon adenocarcinoma	HaCat	HT-29 and SW620	48 h	1 mM	Cell growth (−), G1 arrest (+), p15 and p21protein expression (+), RB protein phosphorylation (−)	-
Paduch et al. [40]	Human colon adenocarcinoma	CCD 841 CoTr	HT29	24 h	100 to 500 μg/ml	Cytotoxicity (+), did not affect the expression of reactive oxygen species (ROS) and IL-6 production	HT29 = 458.3 μg/mL, CCD 841 and CoTr = 273.7 μg/mL
Peffley et al. [41]	Human colorectal cancer		Caco2	16 h	400 μM	Ser65 phosphorylation (−), Thr37 phosphorylation (+)	-
	Human prostate carcinoma		DU145 and PC3			All cells: Ser65 phosphorylation (−), did not affect Thr37 phosphorylation; DU145: Akt phosphorylation (+), PC3: did not affect Akt phosphorylation	
Ma et al. [36]	Human colon carcinoma, human liver adenocarcinoma, and human uterine adenocarcinoma	-	HCT116, SK-Hep1 and HeLa	30 min	20 μM to 200 μM	All cells: HIF-1α reporter gene (−); HCT116: hypoxia-induced ROS (−), expression of phospho-mTOR, phospho-4EBP1, and phospho-eIF4E induced under hypoxia (−), VEGF and EPO mRNA expression (−), VEGF or GLUT-1 protein expression (−)	-
				12 h	200 μM	HCT116: Akt phosphorylation (+), HIF-1α protein levels (−)	
				24 h	20 μM to 200 μM	HCT116: Did not affect cell viability in all cells, G1 phase cell cycle arrest (+)	
Oturanel et al. [39]	Human hepatocellular carcinoma	NIH/3T3	HepG2	24 h	20, 50, 100, 200, and 500 μg/mL	Cytotoxicity (+), DNA synthesis (−)	NIH/3T3 = 250 μg/mL, A549 > 500 μg/mL and HepG2 = 409.2 μg/mL
				24 h	409.2, 500, and 38.4 μg/mL	Apoptosis (+), caspase 3 (+), no effect on depolarization of mitochondrial membrane	
	Human lung adenocarcinoma		A549	24 h	20, 50, 100, 200, and 500 μg/mL	Not cytotoxic, DNA synthesis (−)	
				24 h	500, 125, and 21.5 μg/mL	Apoptosis (+), caspase 3 (+), no effect on depolarization of mitochondrial membrane	
de Lima et al. [25]	Human lung cancer	-	H209 and H1734	24 h	500 μM a 1500 μM	Caspase-3 (+), PARP-1 (−), Apoptosis (+)	H209 = 975 μM and H1734 = 725 μM
				48 h	500 a 2000 μM	Cell viability (−)	
			H69, H82, H358 and H2347				
Elegbede et al. [27]	Human lung adenocarcinoma and human tongue squamous cell carcinoma	-	A549 and BroTo	12 h	1 mM to 4 mM	Colony formation (−)	A549 = 1.2 mM and BroTo = 1 mM
				24 h	1 mM	Cell proliferation (−), apoptosis (+), cell cycle arrest in G0/G1 (+)	
da Gama Fischer et al. [28]	Human lung adenocarcinoma	-	A549	48 h	1.8 mM	Cell proliferation (−) and HSP70 expression (−)	-
				Not informed	0.18 mM	p42 phosphorylation (+) and p44 phosphorylation (−)	
Xu et al. [50]	Human bronchioalveolar carcinoma	-	NCI-H322	5 days	0.75 mM	Cell proliferation (−) and colony formation (−)	-
				48 h	1.5 mM	Apoptosis (+), PARP (−)	
				24 h	1.5 mM	Caspase 3 (+)	
				8 h	1.5 mM	H93085-M-phase phosphoprotein 1 (−), AA447662-geminin (−), N47113-PRO2000 proteinn(−), AA676408 (+), AA447746-hypothetical protein (+), T83646-phosphoglycerate dehydrogenase (+), AA644215-JM5 protein (+), A4676804-CCAAT/enhancer binding protein (C/EBP) (+)	
	Human lung adenocarcinoma	-	NCI-H838	8 h	1.5 mM		
				5 days	1.5 mM	Cell proliferation (−) and colony formation (−)	
				72 h	1.5 mM	Apoptosis (+)	
				48 h	1.5 mM	Caspase 3 (+), PARP (−)	
Yeruva et al. [51]	Human lung adenocarcinoma and squamous cell carcinoma	-	A549 and H520	24 h	1 mM	Cell viability (−), sensitivity to cisplatin or radiation (+), G0/G1 arrest in A549 and G2/M arrest in H520 (+)	A549 = 1.4 mM and H520 = 1.7 mM
				24 h	2 mM	Apoptosis (+)	
				24 h	0.5 mM	Bax protein (+), caspase 3 (+), Bcl2 (+) and p21 (+)	
Nandurkar et al. [38]	Human lung adenocarcinoma	-	A549	6 h	400 μM	Cell viability (−) and Ser65/Thr37 phosphorylation (−)	A549 = 350 μM and PC3 = 380 μM
	Human prostatic adenocarcinoma	-	PC3	48 h	250 μM	Cell viability (−)	
Chung et al. [20]	Human prostate carcinoma	-	LNCaP	6 days	0.1 μM to 1 μM	Androgen-induced cell growth (−)	-
				24 h	0.5 μM to 1 μM	Androgen-stimulated secretion of PSA and hK2 protein (−), expression of AR protein (−), c-Jun protein (+), transcriptional activity of the AR gene (−)	
Rajesh and Howard [42]	Human prostate carcinoma	-	DU145 and PC3	72 h	0.3 mM and 0.5 mM	Sensitization of these cells to radiation (+), apoptosis (+), G2/M phase cell cycle (+), G0/G1 phase cell cycle (−), expression of cyclin D1 in the G2/M (+)	-
				24 h	0.1 mM to 0.7 mM	Membrane-bound form of the Fas ligand (+), did not alter the level of expression of the Fas receptor	
				48 h	1 mM	Fas cascade in cell death (+)	
Karlson et al. [33]	Human pancreas epithelioid Carcinoma	-	PANC-1	5 days	0.2 mM	Cell growth (−), did not affect the activity of MAP kinase	-
	12V-H-ras-transformed rat fibroblast cell line	-	A18	5 days	not informed	Cell growth (−)	
Stayrook et al. [48]	Human pancreatic adenocarcinoma	D27	BxPC3, Panc1 and MIA PaCa2	48 h	300 μM	Apoptosis (+), BAK protein (+)	BxPC3 = 200 μM, Panc1 = 215 μM, MIA PaCa2 = 215 μM, B12/13 = 150 μM and D27 = 260 μM
	Hamster pancreatic ductal adenocarcinoma		B12/13	48 h	100–300 μM	Cell growth (−), apoptosis (+)	
				48 h	500 μM	BAK protein (+)	
				60 min	100–300 μM	Did not affect DNA synthesis	
Wiseman et al. [49]	Human pancreatic adenocarcinoma	-	MIA PaCa2	24 h	300 μM	Cell proliferation (−), G0/G1 cell cycle arrest (+)	-
				24 h	300–800 μM	p21 and p27 (+), cyclin D1 (+), cyclin A (−), cyclin B (−), and CDK2 (−), did not affect cyclin E and p57 protein expression, CKI association with CDK2 (+), CDK2 activity (−)	
			BxPC3	24 h	150–600 μM		
				24 h	500 μM	Cell proliferation (−)	
de Saldanha et al. [26]	Human glioblastoma	-	A172	Not informed	0.06 mM	Cofilin (−)	-
Fischer et al. [29]	Human glioblastoma	-	A172	24 h	1.8 mM	Cell proliferation (−), Ras proteins (+), phosphorylation of the ERKs (−), GSK3β phosphorylation (+), β-catenin (−)	-
Garcia et al. [30]	Human glioblastoma	-	A172	30 min	0.25 mM to 5 mM	Na/K-ATPase activity (−)	1.5 mM
Garcia et al. [31]	Human glioblastoma	Primary cultures of mice astrocytes and Vero	U87 and U251	30 min	4 mM	Na/K-ATPase activity (−), cell viability (−)	VERO = 0.9 mM, mouse astrocytes = 1.4 mM, U251 = 1.4 mM and U87 = 1.1 mM
				30 min	1.5 mM	All cells except VERO: JNK1/2 phosphorylation (+), U87: p38 phosphorylation (+)	
				24 h	1.5 mM	U251: IL-8 release (+)	
				24 h	0.5 mM	U87 and U251: Apoptosis (+)	
Marín-Ramos et al. [37]	Human glioblastoma	-	Primary glioblastoma stem-cells (GSCs)	48 h	1 mM to 3 mM	Cytotoxicity (+), did not differentiate the GSCs as did not affect Sox2 expression	1.2 to 1.8 mM
				24 h	1.5 mM	GRP78 (+), CHOP (+), ATF3 (+), PARP (+), cleaved caspase 7 (+), P62 (+), migration and invasion (−) and ER stress-mediated apoptosis (+)	
				24 h	100 μM	Adhesion molecules MYH9, TGFB1, ACTN3, ILK, ITGB2, ITGB3, MMP14, RHOA, BCAR1, CAPN1, CAPN2, CAV1, and ENAH (−); factors involved in cellular projections ACTR3, CFL1, CTTN, MMP2, MYH10, PIK3CA, PLD1, RND3, SH3PXD2A, and WASF2 (−); factors related to the Rho family of GTPases LIMK1 and PTPN1 (−); EGF and IGF1 (−)	
				48 h	100 μM	Levels of RhoA (+), formation of pro-migratory structures (−), phosphorylation of Src (−), phosphorilation of p42/44 MAPK, Akt, and Stat3 (−)	
Rajesh et al. [43]	Human glioblastoma	-	T98G	72 h	0.7 mM and 1 mM	Cell viability (−)	-
				8 h	0.5 mM	Radiosensitization (−)	
				24, 48 and 72 h	0.5 mM and 1 mM	sub-G0/G1 population (+)	
				72 h	0.5 mM and 1 mM	Cell cycle G2/M phase (+) and G0/G1 phase (−)	
				48 h	0.3–1 mM	Block in the G2/M phase	
				72 h	0.1–0.3 mM	Membrane-bound Fas-L (+)	
				48 h	0.5 mM and 1 mM	Fas cascade in cell death (+)	
				36 h	0.1–0.5 mM	Sensitization to cell death induced by cisplatin and doxorubicin (+)	
				72 h	0.1–0.5 mM	Caspase 8 (+)	
	Human glioblastoma, Astrocytoma and rat glioma		MO59K, U87, U251, U373 and C6	72 h	0.1–0.5 mM	All cells: G2/M phase of the cell cycle (+), expression of the membrane-bound form of the Fas-L (+), Fas (+), sensitized toFas mediated apoptosis (+); U251 and C6: radiosensitization (+), U87: Fas receptor (+)	
Shi and Gould [45]	Mouse neuroblastoma	-	Neuro-2A	12 h	1 mM	Neurite outgrowth (+)	-
				45 h	1 mM	DNA synthesis (−), cell proliferation (−), ubiquinone (CoQ) synthesis (−)	
Silva et al. [47]	Medulloblastoma	-	DAOY, ONS76, UW402 and UW473	48 h	0.5 mM to 8 mM	Increase in necrotic cells, did not change caspase-3 activation	DAOY = 0.74 mM, ONS-76 = 0.76 mM, UW402 = 1.92 mM, and UW473 = 2.01 mM
				-	IC50 values	ONS76 and DAOY: ERK1/2 phosphorylation (−); UW402 and UW473: ERK1/2 phosphorylation (+)	
Shi and Gould [46]	Murine transformed mammary epithelial	-	TM6	20 h	0.5 mM	Cell proliferation (−)	-
				15 h	0.5 mM	G2/M and G0/G1 cell cycle arrest (+), pRB phosphorylation (−), cyclin D1-associated kinase and the cyclin E-associated kinase activity (−), cyclin D1 protein levels (−), did not affect levels of cyclin E and CDK4 protein, p21 (+), p21 association with cyclin E-CDK2 (+), did not affect the p21 association with cyclin D1-CDK, cyclin E-CDK2 kinase activity (−), pRB phosphorilation (−), cyclin A protein (−)	

## Data Availability

The original contributions presented in this study are included in the article/Supplementary Materials; further inquiries can be directed to the corresponding author.

## Supplementary Materials

The following supporting information can be downloaded at: https://www.mdpi.com/article/10.3390/cancers16234003/s1.
